# A virtual reality-based multicomponent intervention program for preventing postoperative delirium in the intensive care unit for cardiac surgery patients: from evidence-based design to a pilot trial

**DOI:** 10.3389/fmed.2026.1815659

**Published:** 2026-06-02

**Authors:** Jing Dong, Qiqi Ni, Xiaoyan Gong, Weijing Sui, Li Wang, Zhenzhen Huang, Jia Yi, Ran Yan, Weilin Jiang, Hongting Zhou, Junru Zhang, Yiyu Zhuang

**Affiliations:** 1 Department of Nursing, Sir Run Run Shaw Hospital, Zhejiang University School of Medicine, Hangzhou, China; 2 Zhejiang University School of Medicine, Hangzhou, China

**Keywords:** cardiac surgery, critical care, pilot study, postoperative delirium, virtual reality

## Abstract

**Background:**

Postoperative delirium (POD) after cardiac surgery in the intensive care unit (ICU) has a high incidence and poor outcomes. Virtual reality (VR) is a non-pharmacological intervention of growing interest, but its effects in cardiac surgery patients remain underexplored.

**Aim:**

To evaluate the feasibility of a perioperative VR-based intervention program and to explore its preliminary clinical effects on POD, anxiety, depression, cognition, sleep, and mobility in cardiac surgery patients.

**Study design:**

A randomized controlled trial was conducted from February to March 2025 in a tertiary hospital in China. Forty participants undergoing elective cardiac surgery were assigned to usual care (control group) or usual care plus daily VR sessions from preoperative preparation until ICU discharge (intervention group) (*n* = 20 each). Outcomes included delirium incidence, severity, duration, onset time, and changes in stress, cognition, sleep, and mobility with time perioperatively.

**Results:**

In this pilot trial, the intervention group had a lower incidence of delirium than the control group (5% vs. 35%, *p* = 0.048). However, the study was not powered to detect differences in delirium incidence, and the limited number of events (n = 8) makes this estimate unstable. Therefore, this finding should be interpreted as a hypothesis-generating signal only, not as evidence of clinical efficacy. The intervention group also had lower delirium severity, higher Barthel Index scores, and shorter ICU length of stay (all *p* < 0.05). In addition, patients in the intervention group had lower stress levels at multiple perioperative time points, better sleep on the first and last ICU nights, and higher cognitive scores at ICU discharge than those in the control group. Repeated-measures analyses suggested favorable changes over time in anxiety, depression, and sleep in the intervention group.

**Conclusion:**

Perioperative VR-based multicomponent intervention was feasible and well accepted in this pilot study, and may indicate potential benefit in reducing POD and improving selected psychological and functional outcomes in cardiac surgery patients. These findings are preliminary and require confirmation in larger, adequately powered multicenter trials.

## 1 Introduction

Cardiovascular disease remains a leading global health threat in the 21st century (1–3), with over one million cardiac surgeries performed annually worldwide (4). As a high-risk procedure, cardiac surgery is associated with postoperative complication rates as high as 60% (5), among which delirium is one of the most common (6), with an incidence of 23–52% during ICU stays (7–9). Postoperative delirium (POD), typically occurring 24–72 h after surgery, is characterized by acute cognitive disturbances, including inattention, disorganized thinking, and fluctuating consciousness (10–12). Its incidence is influenced by factors such as surgical trauma, extracorporeal circulation, and ICU admission, raising the risk by 9–73% (9, 13, 14). The ICU environment itself—marked by stress, sleep disruption, and sensory imbalance—further contributes to delirium risk (6, 15–17). Beyond its high prevalence, POD is associated with increased complications, mortality, readmissions, healthcare costs, and long-term impairments in physical, cognitive, and psychological functioning, ultimately reducing patients’ quality of life (18–20).

For POD, prevention is currently the first choice for patients admitted to the ICU after cardiac surgery (6, 21, 22). As noted in the guidelines, it is essential to implement early preventive interventions for POD after cardiac surgery (10, 23, 24). Currently, there is insufficient pharmacological evidence for prevention. Non-pharmacological interventions have been recommended as the preferred means of preventing POD (7, 10, 25), which are designed to reduce one or more modifiable risk factors for POD (9, 15, 26), are easier to implement, and are more cost-effective. Notably, although some non-pharmacological prevention methods, such as ABCDEF bundled care measures, early activity, cognitive training, preoperative education (7, 27–31), as well as multisensory stimulation interventions focused on psychological support and sleep in several recent studies have demonstrated partial clinical benefit and potential value (30, 32), the direct effect on delirium prevention is still uncertain (33, 34). Thus, POD prevention after cardiac surgery is still challenging (35). Meanwhile, although nurse-led non-pharmacological interventions have been established as a first-line approach for POD prevention, their clinical application in cardiac surgery patients still faces multiple practical barriers. On the one hand, the multicomponent prevention strategies recommended by guidelines are often poorly integrated in routine care, with individual elements delivered in a fragmented manner that fails to achieve synergistic effects (36). On the other hand, conventional non-pharmacological interventions are mostly fixed in their design, making it difficult to adapt to the difficulty or content in real time, thereby limiting their ability to accurately respond to patients’ constantly changing clinical needs (37, 38). The intervention delivery process itself also suffers from insufficient standardization; the content and intensity of interventions may vary considerably depending on the experience and workload of individual nurses (39, 40). In addition, the ICU environment offers little controllability—patients receiving usual care remain continuously exposed to adverse stimuli such as monitor alarms, nocturnal light exposure, and frequent care interruptions, which may counteract the intended benefits of preventive strategies (41). Patient engagement is another major concern (42), limited by the vulnerability of the cardiac surgery population and the specificity of the ICU environment to which they are transferred postoperatively (41), these nurse-led non-pharmacological preventive strategies are unevenly implemented, the intervention approach is highly dependent on the personal intervention of healthcare professionals (40, 43), with low patient compliance, autonomy, and motivation to participate, and the range and intensity of the stimuli are limited (44), making the intervention’s implementation likely to be ineffective. Thus, what is truly lacking is not the concept of prevention but rather an intervention tool that can integrate multicomponent content, provide dynamic adaptability, enable standardized implementation, enhance patient participation, create a controllable intervention environment that avoids adverse stimuli, and ultimately align with the perioperative clinical context of cardiac surgery patients. It is evident that in the current era of rapidly evolving digital health technologies (45, 46), traditional or non-interactive preventive measures may not meet patients’ needs and expectations. Given the current barriers and limitations of clinical implementation of non-pharmacological prevention strategies, developing advanced technologies such as virtual reality (VR) may offer useful directions for POD prevention (47).

Virtual reality technology uses sensors, multimedia, computer simulation, computer graphics, and other technologies to create a three-dimensional, dynamic view of the virtual world, allowing patients to be immersed in it and achieve human-computer interaction (48, 49). Virtual reality technology not only has a high degree of immersion, interactivity, conceptualization, and guided active engagement, but also has more time- and space-independent mobile high-performance, lightweight advantages (50), which is conducive to its being integrated and applied in routine clinical environments (51). In clinical populations, including cardiac surgery patients, virtual reality technology has shown clinical effects in stress relief, sleep promotion, neu-cognitive stimulation, early motor rehabilitation, and other components conducive to preventing delirium development (52–54). Accordingly, a few studies have tried to explore the effect of non-pharmacological strategies implemented with the help of virtual reality technology on delirium prevention (55–57). Still, none of the current findings have confirmed its direct effectiveness on delirium prevention. The study population was relatively broad, with a single intervention content that did not consider the advantages of multicomponent intervention strategies for delirium prevention (6, 7). Preoperative anxiety and sleep disturbances in patients are associated with adverse outcomes such as POD (58–61). Therefore, besides the above postoperative multicomponent intervention components, recent studies have shown that applying a virtual reality exposure experience as preoperative education effectively reduces preoperative anxiety in adult patients undergoing elective cardiac surgery (33, 62–65). Furthermore, preoperative education with virtual reality simulation of the postoperative ICU stay experience has also been found to improve outcomes for cardiac surgery patients, including both preoperative and postoperative anxiety. Still, its impact on the incidence of POD remains to be further demonstrated (66). Based on the stated experiences and clinical needs of patients with POD in previous studies (67, 68), patients felt that being provided with detailed information about the ICU and POD preoperatively would be meaningful in preventing or reducing the occurrence of their POD. Therefore, using virtual reality to provide visual and more understandable preoperative education to deliver virtual ICU visits, including introducing POD, may offer potential advantages. The above evidence suggests that the implementation of non-pharmacological multicomponent prevention strategies based on virtual reality technology, including modular components such as virtual ICU visits for preoperative education, psychological, sleep, cognitive, and early motor rehabilitation, may be a potentially useful approach for preventive interventions for POD after cardiac surgery in the ICU. However, as mentioned earlier, research on virtual reality interventions specifically targeting POD prevention in cardiac surgery patients remains scarce. Existing studies have primarily focused on single-component interventions and have not fully leveraged the potential of VR to deliver integrated multicomponent strategies covering psychological, sleep, cognitive, and motor domains (66, 69). Consequently, robust empirical evidence from well-designed clinical studies is still lacking, underscoring the need for rigorous investigations that evaluate multicomponent VR-based interventions in this high-risk population.

Therefore, given the lack of virtual reality-based multicomponent delirium preventive interventions tailored for cardiac surgery patients, and the preliminary evidence suggesting possible benefit, the research team developed a virtual reality-based system for preventing POD and a structured intervention program for cardiac surgery, which was based on previous studies and guidelines (10, 67, 70) and combined theories such as stress recovery theory and attention recovery theory (71, 72).

## 2 Aims and objectives

The primary objective of this double-arm pilot randomized controlled trial is to assess the feasibility and explore the preliminary clinical effects of the virtual reality-based multicomponent intervention for POD prevention in patients undergoing elective cardiac surgery. And to explore the initial impact of the program on patients’ psychology (anxiety, depression levels), sleep, cognition, mobility, and other relevant clinical outcomes (e.g., ICU length of stay). The results of this pilot study are a foundational step to support future investigations of the efficacy and safety of virtual reality-based multicomponent interventions in preventing POD after cardiac surgery in more centers and larger samples.

## 3 Design and methods

### 3.1 Study design

This study was a single-center, double-arm, parallel-design, prospective randomized controlled trial, registered in the Chinese Clinical Trial Registry (ChiCTR2400090962) on 16 October 2024. Sequence generation: The allocation sequence was generated using a computer-based random number generator, ensuring an unpredictable and unbiased sequence. Allocation concealment: Allocation was implemented using sequentially numbered, opaque, sealed envelopes (SNOSE) prepared by an independent researcher not involved in participant recruitment or outcome assessment, thereby ensuring adequate concealment and preventing foreknowledge of assignment. Implementation: Participants were enrolled by the research team, and group assignment was revealed only after enrollment by opening the corresponding envelope in sequence. The results were reported according to the Consolidated Standards of Reporting Trials (CONSORT) statement (see Appendix A) (73).

### 3.2 Setting and sample

Participants were recruited from the cardiac surgery department of a tertiary-level general hospital in Hangzhou, Zhejiang Province, China, between February and March 2025. The participants were patients scheduled to undergo elective cardiac surgery and expected to be transferred to the ICU postoperatively.

Inclusion criteria were (1) age ≥18 years; (2) undergoing elective open-heart surgery under general anesthesia with extracorporeal circulation and anticipating postoperative transfer to the ICU, with types of surgery including heart valve surgery, coronary artery bypass grafting, and major vascular surgery; (3) with an American Society of Anesthesiologists (ASA) Physical Condition Classification System rating of I to II; and (4) had not undergone cardiac surgery or had a history of ICU admission within the past 30 days.

Exclusion criteria were (1) impending emergency surgery; (2) having severe cognitive impairment or dementia or psychiatric disorders or other severe brain dysfunction affecting delirium assessment; (3) having used psychotropic medications or having a history of alcohol or drug abuse; (4) having severe hearing, vision, or speech impairments preventing textual or verbal communication; (5) having vestibular dysfunction or dyscalculia; (6) having a history of psychological disorders or severe sleep disorders, or neurological disorders such as Parkinson’s disease, myasthenia gravis, multiple sclerosis, muscular dystrophy; (7) inability to learn and cooperate with the use of a virtual reality; (8) scheduled for a second surgery within 7 days of the procedure; and (9) participating in other clinical trials.

Withdrawal criteria were: (1) surgery being cancelled after randomization or withdrawal before the first postoperative assessment; (2) dying before intraoperative or postoperative evaluation; (3) not being transferred to the ICU as planned after surgery or having an ICU stay of less than 24 h; and (4) persistent coma or need for deep sedation due to the condition, i.e., persistent ≤ − 3 points on the Richmond Agitation-Sedation Scale (RASS) score after the anesthesia resuscitation period.

Dropout criteria: (1) withdrawal from the study during the postoperative intervention in the ICU for various reasons (active abandonment, serious condition, adverse reactions) and (2) loss of visits after the postoperative intervention (early ICU discharge).

The sample size was determined by referencing the recommended sample size for pilot studies in previous scientific literature, with scholars suggesting a sample size of 10–40 cases for pilot studies (74, 75). Considering the above calculation methods, the research team’s human and material resources, and the potential dropout rate, the sample size for this pilot study was determined to be 40 cases (20 cases each in the intervention and control groups).

### 3.3 Recruitment, randomization, and blinding

The principal investigator screened eligible potential participants from patients in cardiac surgery who had already been scheduled for surgery based on inclusion and exclusion criteria. The researchers gave them a detailed explanation of the study content and potential risks. Each participant was fully informed and given sufficient time to consider whether to participate and provide consent. Participants were told the right to withdraw without any consequences.

A research assistant not involved in data collection generated a random list of numbers from 1 to 40, divided into two groups of 20 cases each. A ‘unique’ random number generator was used to avoid duplicate numbers, with random numbers 1–20 assigned to the control group and others to the intervention group. The study coordinator placed the group assignment information into opaque, sequentially coded envelopes and sealed them. These numbers were printed separately and were only accessible to the study coordinator during the randomization process. After screening and confirming participant eligibility, the principal investigator inquired about group assignment from the research assistant. The research assistant then sequentially opened the next randomization envelope according to the order of patient enrollment and determined the group assignment of patients based on the allocation scheme inside the envelope, randomly assigning eligible participants to one of the two groups. Randomization was conducted at the start of the study. Due to the nature of the intervention measures, it was difficult to blind the patients and intervention investigators. In this study, the outcome assessors and statistical analysts were blinded; they did not participate in other aspects and were unaware of the entire study protocol and patient grouping.

### 3.4 Procedure

#### 3.4.1 Establishment of the research team

A multidisciplinary research and development team was established, which comprised seven researchers (two nursing management specialists, one PhD candidate in nursing science, one specialist nurse each from cardiac surgery and ICU, one physician each from cardiac surgery and ICU, one registered nurse with mindfulness and meditation experience holding a psychological counsellor qualification, and one critical care rehabilitation technician) and five software development engineers from a virtual reality technology company. It should be specifically noted that a critical care rehabilitation technician was part of the research team. Their primary responsibilities included: (1) co-designing the safety parameters and functional movement sequences for the VR-based limb exercise module; (2) training the research nurses on principles of safe patient mobilization and the correct facilitation of the conventional nurse-assisted mobility provided to the control group; and (3) being available for consultation regarding any mobility-related safety concerns during the study. The technician was not directly involved in the day-to-day, patient-facing delivery of either the VR-delivered multicomponent intervention or the control group’s mobility sessions, ensuring that the interventions were delivered consistently by the nursing staff.

#### 3.4.2 Program development

Based on an evidence-based framework, we developed a virtual reality-based system for preventing POD after cardiac surgery. The software was implemented using Unity 2022 as the development platform, with C# as the programming language. The virtual reality system comprises the following modules: (1) Preoperative Education (Preoperative Virtual ICU Visit): Providing a simulated ICU experience preoperatively. The virtual visit includes introducing treatment, the nursing process, and POD. (2) Stress Response Mitigation (Natural Scene Meditation): Providing immersive natural environments through sensory stimulation, with dynamic adjustment of scene elements (e.g., light intensity, wind speed) and gentle female-voiced mindfulness meditation. (3) Circadian Reorientation (Sleep Environment Simulation): Creating immersive sleep scenarios such as interstellar travel, accompanied by hypnotic music. (4) Cognitive Stimulation (Interactive Cognitive Training Games): Implementing cognitive rehabilitation training through virtual scenarios, targeting attention, memory, calculation, executive function, and recall abilities. (5) Early Mobility Promotion (Interactive Virtual Physiotherapy): Developing virtual reality games for early exercise, incorporating inertial sensors for millimeter-level motion capture. A detailed description of the content, technical parameters, and progression logic for each of the five VR modules is provided in Appendix B: Supplementary Table S1 (Page 66–67).

**Figure 1:**
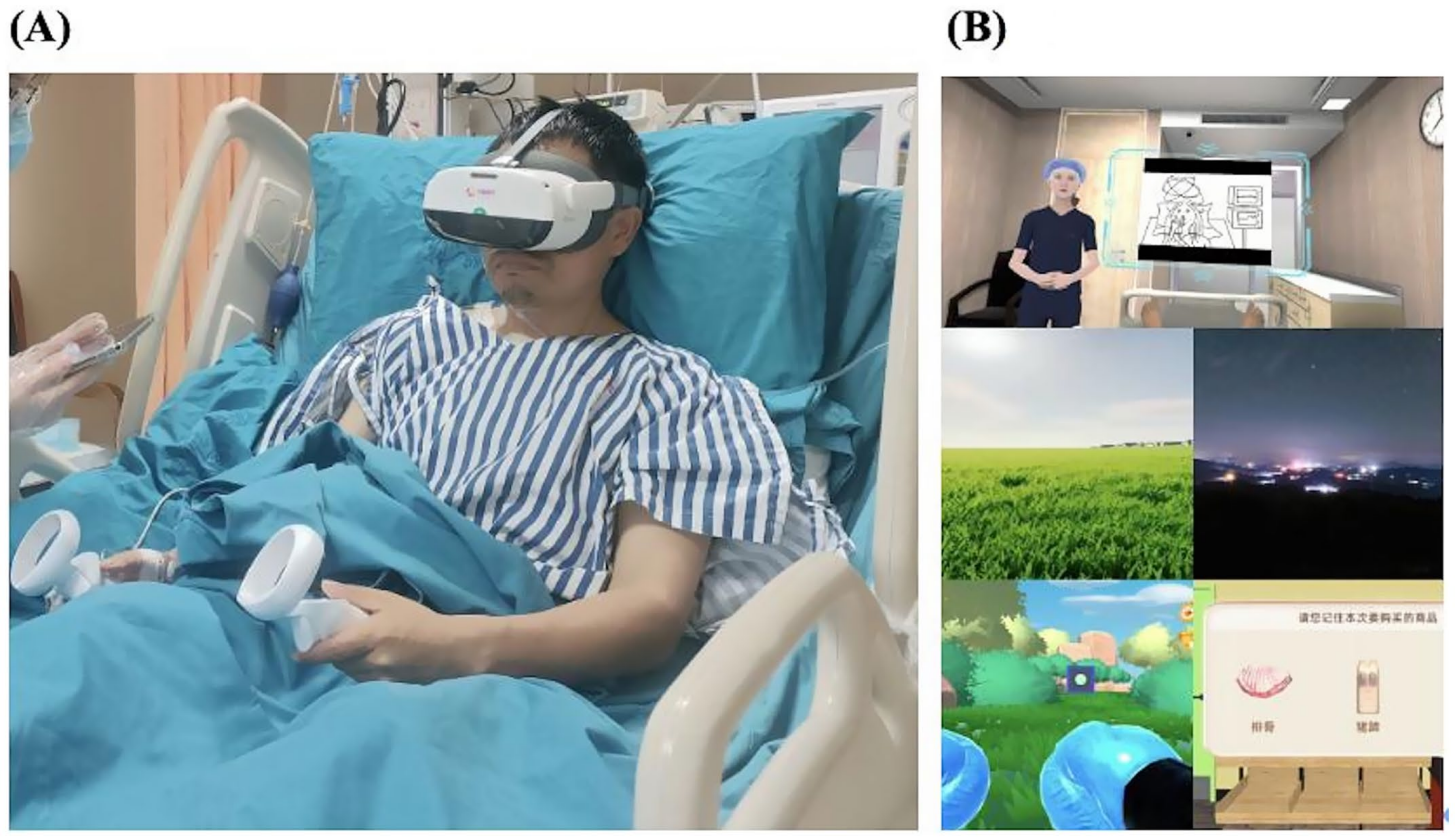
Overview of virtual reality hardware and intervention. **(A)** This image shows a patient using the Pico Neo3 virtual reality standard edition kits (including a head-mounted display, two wireless controllers, and an adjustable head strap). **(B)** Representative screenshots of the five core VR-delivered multicomponent intervention modules. (i) Preoperative ICU Visit: A 3D-rendered simulation of a typical intensive care unit (ICU) bed space, featuring virtual medical equipment (e.g., monitor, ventilator) to familiarize patients with the postoperative environment.(ii) Nature Scene Meditation: A serene, immersive 360° natural environment (e.g., forest) used as a backdrop for audio-guided mindfulness and relaxation exercises. (iii) Sleep Environment Simulation: A “Stargazing” scenario, showing a static, high-resolution panoramic view of a starry night sky designed to promote circadian reorientation and relaxation. (iv) Cognitive Training Game: A first-person view of a gamified cognitive task (e.g., a virtual Stroop test) where users select targets to train attention and executive function. (v) Virtual Physiotherapy: A screenshot from a guided upper-limb exercise module, where a patient’s virtual hands are shown interacting with objects (e.g., reaching for a bottle) to promote early functional mobility. All screenshots are sourced directly from the custom-developed VR application.

The hardware comprises Pico Neo3 virtual reality Standard Edition kits (a head-mounted display, two wireless controllers, and an adjustable head strap). The head-mounted display integrates front-facing cameras, stereo speakers, and a microphone. PADs (Lenovo) were provided for control purposes. Replaceable face covers were procured to minimize cross-infection. The overview of virtual reality hardware and intervention is in Figure 1.

#### 3.4.3 The intervention and control groups

(1) The intervention group

The intervention group added a virtual reality-based multicomponent intervention to usual care. The intervention relied on the above self-developed system, including five modules. For patients with scheduled cardiac surgery dates, a preoperative virtual ICU visit intervention was conducted using virtual reality in the cardiac surgery department one day before surgery, lasting 5–6 min. After the patient was transferred to the ICU and had completed the anesthesia recovery period, the following interventions were conducted daily until ICU discharge: At 09:00 during the day shift, patients underwent virtual reality-based interactive cognitive training and virtual limb exercise interventions, with each module lasting approximately 10–15 min. At 16:30 during the day shift, patients received a virtual reality-based natural scene meditation intervention, lasting approximately 20–25 min. At approximately 20:30 during the evening shift, a virtual reality-based rhythmic sleep environment is created for the patient approximately 20–30 min before bedtime (can be adjusted flexibly based on actual condition), lasting approximately 10–15 min. Six nursing staff members undergoing virtual reality operation training implemented the interventions.

Furthermore, to control cross-infection between patients, six VR headsets (Pico Neo 3) were used in the study, and each was equipped with six replaceable foam paddings for hygiene purposes. Each headset and its controllers were assigned to a single patient for the duration of their ICU stay. Between patients, all hardware components were thoroughly cleaned and disinfected using 75% medical-grade alcohol wipes, followed by exposure to ultraviolet-C light in a dedicated disinfection cabinet for 30 min. This protocol was reviewed and approved by the hospital’s Infection Control Department.

(2) The control group

The control group received usual care, primarily implemented by nurses. On the day before surgery, the on-duty nurse conducted usual preoperative education (verbal or cell phone video) for approximately 10 min. After surgery, under the guidance of ICU nurses, the following bundle care measures were implemented: nurse-guided numerical games; providing earplugs and eye masks; nurses assisted patients with active and passive mobility; using Bluetooth speakers to play relaxing music; nurses used magnifying glasses and other audio-visual equipment to communicate with patients to prevent or treat sensory deprivation. Bundle care interventions began when the patient was transferred to the ICU and, after the anesthesia resuscitation period, were performed daily until ICU discharge. All nurses involved in delivering the control interventions received standardized training on the routine delirium prevention protocol before the study commenced. The interventions followed the hospital’s established clinical pathway for cardiac surgery ICU patients. Although the interventions were standardized through training and a clinical pathway, the exact dose, frequency, and consistency of each component were not systematically logged or audited. Intervention fidelity may have varied among nursing staff, which represents a potential source of bias.

(3) If a patient was diagnosed with delirium by CAM-ICU on a given day, the intervention would stop until re-diagnosed as delirium-negative by CAM-ICU. At this point, the intervention resumed, and the cycle repeated. Intervention records were documented each time in a unified electronic spreadsheet, facilitating real-time coordination and tracking by the research team managers. Interventions were conducted under the supervision of researchers and professional medical staff. If any discomfort was observed in a participant, the intervention was immediately terminated, and medical care and support were provided. Researchers checked that tubes were securely fixed and that the bedrails were raised before intervention, and closely monitored during the intervention. Each virtual reality intervention session was strictly controlled, not to exceed 30 min.

### 3.5 Data collection tools and methods

#### 3.5.1 Data collection tools

##### 3.5.1.1 Baseline data

Baseline data include demographic and clinical information. Demographic information included gender, age, education level, marital status, occupation, residence condition, Body Mass Index (BMI), personal history, cardiac surgery or ICU admission history before the last 30 days, and virtual reality experience. Clinical information included surgery type and duration, cardiac function classification, Acute Physiology and Chronic Health Evaluation II (APACHE-II), Sequential Organ Failure Assessment (SOFA), medical history, ICU ward types, mechanical ventilation duration, and medication use. Researchers collected all data after obtaining informed consent from patients or their surrogate decision makers, with some data obtained from electronic medical and nursing records.

##### 3.5.1.2 Feasibility

(1) System usability scale (SUS)

The scale was used to measure the usability of electronic devices, developed by John Brooke et al. (76). The scale consists of ten items across two dimensions: Learnability (2 items) and Usability (8 items). A 5-point Likert scale was used. A total score of >70 suggests good usability. In this study, the Cronbach’s *α* for the learnability dimension, usability dimension, and total scale were 0.742, 0.821, and 0.795.

(2) Igroup presence questionnaire (IPQ)

This scale was used to assess participants’ sense of presence in virtual environments, developed by Schubert et al. (77). The scale has three dimensions: Spatial Presence, Involvement, and Experienced Realism, and a General Presence dimension. A 7-point Likert scale was used. A higher total score indicates a higher level of immersion. The Cronbach’s α of this scale is 0.757 in the Chinese population (78), which is 0.712 in the present study.

(3) Simulator sickness questionnaire (SSQ)

This questionnaire is a subjective tool for assessing the severity of disease symptoms in simulators (e.g., virtual reality devices), developed by Kennedy et al. (79). The SSQ consists of 16 items divided into three symptom categories: nausea symptoms, oculomotor nerve disorders, and disorientation. The severity of each symptom is scored on a scale of 0–3. A higher total score indicates a more severe degree.

##### 3.5.1.3 Primary outcome

(1) Confusion assessment method for the intensive care unit (CAM-ICU)

CAM-ICU was developed by Ely et al. (80) based on the gold standard for delirium diagnosis—DSM-5. Delirium assessment can begin when the RASS score is ≥ − 3 points. The assessment process is divided into four aspects: (1) Acute changes or fluctuations in consciousness, i.e., changes in RASS scores within 24 h. (2) Attention deficits, assessed using the digit method to determine whether the patient can concentrate and make correct judgments. (3) Changes in consciousness level, i.e., RASS score not equal to 0. (4) Disorganized thinking, assessed by questioning the patient to determine if their thinking is normal. If both criteria (1) and (2) are positive, plus either criterion (3) or (4) is positive, a positive CAM-ICU diagnosis is made, indicating the presence of delirium.

##### 3.5.1.4 Secondary outcomes

(1) Confusion assessment method for the ICU-7 delirium severity scale (CAM-ICU-7)

The scale is a quantitative assessment tool developed by Khan et al. (81) based on the CAM-ICU scale, and adapted to the Chinese context by Li et al. (82) in 2022. The scale includes four items: acute onset or fluctuation of mental status, inattention, altered level of consciousness, and disorganized thinking. The total score is 0, indicating no delirium, 3–5, mild to moderate delirium, and 6–7, severe delirium. The Cronbach’s *α* for this scale is 0.85, and 0.82 for the Chinese version.

(2) Hospital anxiety depression scale (HADS)

The HADS scale was developed by Zigmond et al. (83), commonly employed to assess patients’ psychological state. This scale includes 14 items, scored using a 4-point Likert scale. The total score ranges from 0 to 21, with higher scores indicating more severe anxiety or depression. The Cronbach’s *α* in the Chinese population is 0.879.

(3) Mini-mental state examination (MMSE)

The scale was developed by Folstein et al. (84) in 1975 and adapted into Chinese by Zhang et al. (85). It is one of the most widely used cognitive function screening scales. It consists of 30 items. If the participant answers correctly or performs the task correctly, each item is scored 1 point; otherwise, it is scored 0. The total score ranges from 0 to 30 points, with higher scores indicating better cognitive function. The Cronbach’s α for the Chinese version is 0.55 to 0.91.

(4) Richards-Campbell sleep questionnaire (RCSQ)

This scale was developed by Richards et al. (86) to assess patients’ subjective sleep, consisting of five items. The visual analog scale (VAS) scoring method is used. Zero represents the worst, and 100 represents the best. The total score is the average of the five items. Higher total scores indicate better sleep quality. The Cronbach’s α for this scale is 0.89–0.92 (87, 88), also high in the Chinese population, with a value of 0.874–0.895 (89, 90).

#### 3.5.2 Data collection methods

Demographic and clinical information: The researchers collected demographic and clinical information preoperatively at the start of the assessment. The remaining clinical data (e.g., APACHE-II score) were collected from the electronic medical and nursing systems after the patient was discharged from the ICU.

Feasible assessment: Assessed after experiencing all modules within the virtual reality system at least once each.

Delirium-related data: Delirium and its severity were screened for, respectively, using the CAM-ICU tool and CAM-ICU-7 scale by experienced ICU specialist nurses at the time of ICU admission (after awakening from anesthesia), and daily at fixed times in the morning and evening. These results were recorded electronically. If a patient is diagnosed as delirium-positive, the frequency of assessments is appropriately increased. Delirium duration was calculated based on the total sum of all delirium-positive periods. The time to first delirium postoperatively was from the time the patient completed cardiac surgery until the first diagnosis of delirium. The Barthel Index was used as a reference for mobility. Furthermore, all potential participants were screened for pre-existing delirium or cognitive impairment during the preoperative visit. This was achieved through a review of medical records and a brief interview conducted by a research nurse, confirming the absence of a documented history of dementia, delirium, or acute cognitive decline. Only patients deemed delirium-free preoperatively were enrolled. However, these CAM-ICU assessments were part of routine clinical care and were not performed as a study-specific, standardized baseline assessment at a fixed preoperative time point. The absence of a research-dedicated standardized baseline delirium and cognitive assessment represents a methodological limitation.

HADS-A and HADS-D scores: Data were collected on 1 day before surgery, at the start of assessment (T0), on the night before surgery after preoperative intervention (T1), within 2 h of the patient regaining consciousness after ICU admission postoperatively (T2), and within 2 h before ICU discharge (T3).

MMSE scores: Data were collected on 1 day before surgery, at the start of assessment (T0), within 2 h of the patient regaining consciousness after ICU admission postoperatively (T1), and within 2 h before ICU discharge (T2).

RCSQ scores: Data were collected on 1 day before surgery, at the start of assessment (T0), on the day of surgery, before leaving the cardiac surgery department, to assess sleep on the night before surgery (T1), the second day of ICU admission regarding the first night’s sleep in the ICU (T2), and within 2 h before ICU discharge regarding the last night’s sleep (T3).

Regarding the data collection process, it is necessary to make an additional explanation as follows: all patient-reported questionnaires (MMSE, HADS) were administered by a member of the research team who received uniform training. For patients with primary school education or below, the researcher read the questions aloud and explained them in simple, standardized language without leading the patient. Assessments were only conducted when the patient was awake, cooperative, and had a Richmond Agitation-Sedation Scale (RASS) score of 0 (alert and calm) or −1 (drowsy). The RASS score at the time of each assessment was documented. Family members were not involved in questionnaire administration due to the ICU’s restricted visitation policy during the study period.

### 3.6 Data analysis

Data were analyzed using IBM SPSS 31.0 for statistical analysis. (1) Statistical description: categorical variables were expressed as frequency (n) and percentage (%); continuous variables conforming to normal distribution were expressed as mean ± standard deviation (X ± SD), and skewed-distributed measures were described in the form of median (M) and interquartile range (IQR). The Shapiro–Wilk normality test was used to test the normality of the variables, and Levene’s test was used to assess homogeneity of variance. (2) Between-group comparisons: Independent samples t-test was used for continuous variables conforming to normal distribution; the Mann–Whitney U rank-sum test was used for those not normally distributed. Categorical variables were expressed as relative numbers, and dichotomous variables were described by yes or no; according to the theoretical frequency, the Chi-square (χ2) test, Yates’ corrected χ2 test, or Fisher’s exact test were chosen for between-group comparisons. (3) Within-group comparison: Paired samples t-test was used when continuous variables followed a normal distribution, and the Wilcoxon Signed-Rank Test was used for those that did not obey a normal distribution. For normally distributed and variable uniform continuous variables, repeated-measures ANOVA was used for the overall analysis of multiple time points in both groups, and Mauchly’s test of sphericity was used. If it was not fulfilled, Greenhouse–Geisser was used to make corrections. Bonferroni correction was applied to adjust for multiple comparisons within-group changes from T0 to T1, from T1 to T2, and from T2 to T3. Given the large number of secondary outcomes assessed across multiple time points, the risk of type I error (false-positive findings) is increased. Therefore, all secondary outcome findings should be interpreted as exploratory and hypothesis-generating, and are intended primarily to inform the design of future definitive trials rather than to provide confirmatory evidence of efficacy. As this study is a pragmatic trial, data will be analyzed according to the principle of modified intention-to-treat (mITT) to maintain the integrity of the randomization procedure, which means that data will be analyzed for patients who have been randomized, who have received at least one study treatment, and who have the appropriate postoperative efficacy assessment. Statistical significance was set at *p* < 0.05 using a two-tailed test.

### 3.7 Quality control

Quality control was managed by the ICU nurse manager, who monitored the intervention process in each group through on-site inspections, interviews with patients, or review of medical records. (1) The intervention providers were uniformly trained before the intervention, and the intervention was implemented according to a structured standard intervention process to maximize intervention homogeneity. (2) Outcome assessors were not involved in the intervention implementation to minimize measurement bias. Before the study, training for them was uniformly provided, and uniform terminology guidance for questionnaire collection was provided to maximize homogeneity in assessment quality. (3) Data were entered and checked by two persons to minimize data entry errors.

### 3.8 Ethical and institutional approvals

This study was approved by the Clinical Research Ethics Committee on 8 October 2024 (20240555) and in accordance with the Declaration of Helsinki. Researchers received ethics training and signed confidentiality agreements in advance. Written informed consent was obtained from each participant who agreed to participate in the study. The participants have the right to withdraw from the study at any time without any negative impact on their usual treatment, care, and other services. Specifically, before the formal intervention, the researchers fully informed the participants about the study contents, withdrawal, and data confidentiality, and obtained informed consent. Since several subjects of this study were elderly, if participants could not read and sign the informed consent form, their guardians represented and signed the informed consent process. All information related to the identity of the subjects was anonymized and kept confidential by the investigator to protect their privacy strictly.

### 3.9 Trial registration

This study was registered in the Chinese Clinical Trial Registry (ChiCTR2400090962) on 16 October 2024.

## 4 Results

### 4.1 Baseline characteristics of the participants

**Figure 2:**
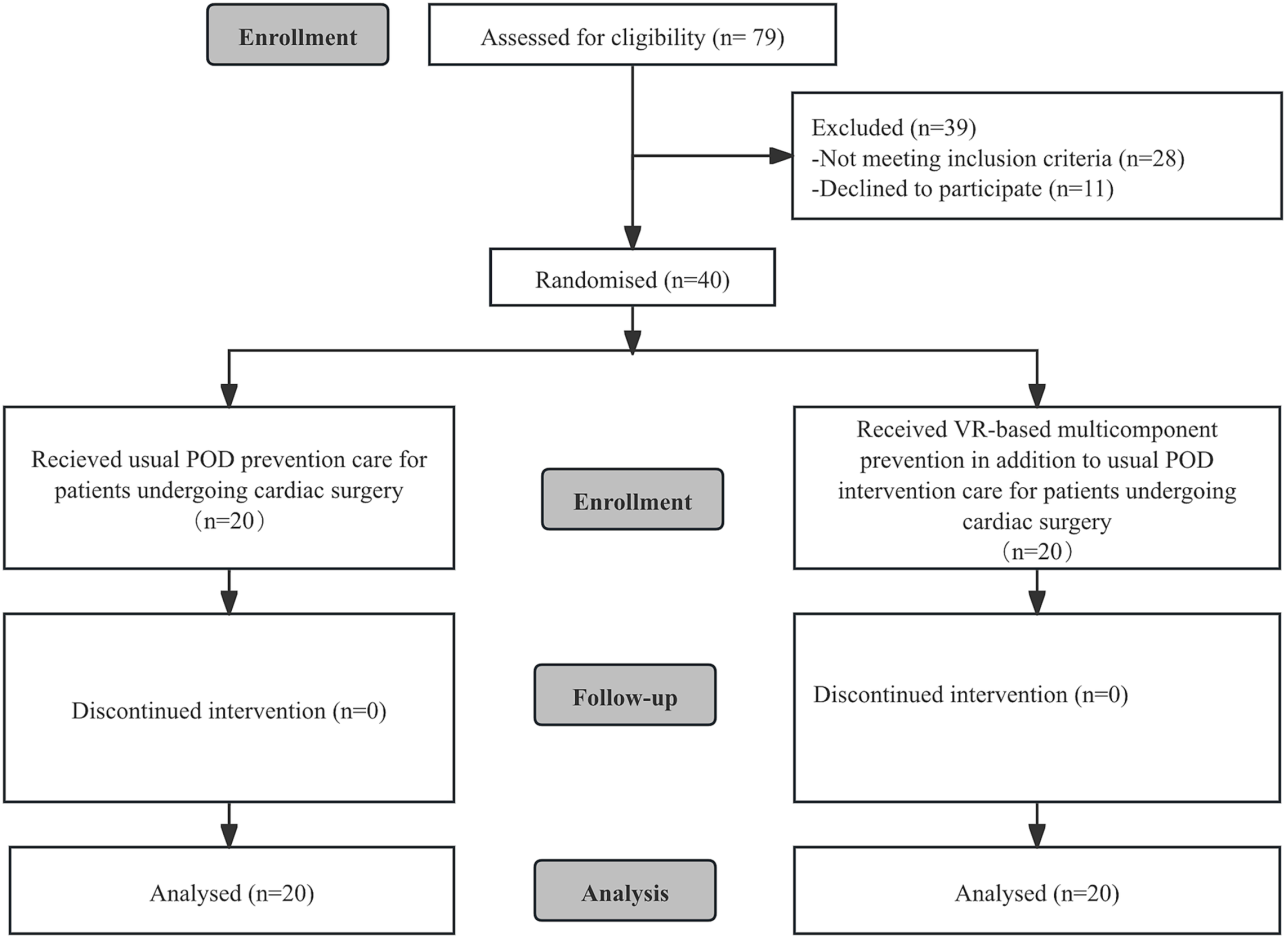
CONSORT flow diagram. CONSORT indicates consolidated standards of reporting trials. POD: postoperative delirium; VR: virtual reality.

Between February and March 2025, 40 of 79 screened patients were randomized equally to the intervention and control groups. All patients completed the intervention process for the full planned number of sessions, with no patients dropping out or missing visits. Table 1 shows that the baseline characteristics, including VR knowledge and demographic/clinical variables, were comparable between groups (all *p* > 0.05). Recruitment details are shown in Figure 2.

**Table 1:** Baseline data of the participants (*N* = 40).

Variables	Intervention group (*n* = 20)	Control group (*n* = 20)	*t*/*χ*^2^/*F*	*p*
Gender, *n* (%)			0.114^a^	0.736
Male	13 (65)	14 (70)		
Female	7 (35)	6 (30)		
Age (years)	64.55 ± 8.58	65.40 ± 7.32	0.337^b^	0.738
Education level, *n* (%)			0.618^d^	>0.999
Primary school and below	9 (45)	9 (45)		
Junior high school	7 (35)	8 (40)		
Senior high school/vocational school	3 (15)	2 (10)		
Junior college or Bachelor’s degree	1 (5)	1 (25)		
Master’s degree and above	0	0		
Marital status, *n* (%)			0.000^c^	>0.999
Married	18 (90)	18 (48.0)		
Unmarried	0	0		
Divorced/ Widowed	2 (10)	2 (10)		
Occupation, *n* (%)			2.849^a^	0.091
Employed	9 (45)	4 (20)		
Unemployed or retired	11 (55)	16 (80)		
Place of residence, *n* (%)			1.758^a^	0.185
Rural areas	15 (75)	11 (55)		
Towns	5 (25)	9 (45)		
Living arrangements, *n* (%)			0.000^c^	>0.999
Alone	1 (5)	2 (10)		
Living with others	19 (95)	18 (90)		
BMI (kg/m^2^)	24.29 ± 3.86	23.12 ± 3.02	–1.068^b^	0.292
Smoking history, *n* (%)			0.440^a^	0.507
Yes	6 (30)	8 (40)		
No	14 (70)	12 (60)		
Drinking history, *n* (%)			0.114^a^	0.736
Yes	6 (30)	7 (35)		
No	14 (70)	13 (65)		
Cardiac surgery or ICU admission history prior to the last 30 days, *n* (%)			0.000^c^	>0.999
Yes	4 (20)	4 (20)		
No	16 (80)	16 (80)		
Having heard of or used virtual reality, *n* (%)			0.000^c^	>0.999
Yes	2 (10)	3 (15)		
No	18 (90)	17 (85)		
Type of surgery, *n* (%)			1.589^d^	0.902
Congenital heart disease surgery	0	0		
Heart valve surgery	6 (30)	7 (35)		
Coronary artery bypass grafting	7 (35)	4 (20)		
Great vessel surgery	4 (20)	5 (25)		
Heart valve surgery + coronary artery bypass grafting	1 (5)	2 (10)		
Others	2 (10)	2 (10)		
Duration of surgery	275.50 ± 86.92	250.25 ± 79.89	-0.956^b^	0.345
Extracorporeal circulation time	143.15 ± 43.23	137.25 ± 39.85	-0.449^b^	0.656
Aortic occlusion time	92.20 ± 37.41	86.85 ± 35.87	-0.462^b^	0.647
Cardiac function classification, *n* (%)			0.383^d^	>0.999
I	1 (5)	1 (5)		
II	10 (50)	11 (55)		
III	9 (45)	8 (40)		
IV	0	0		
APACHE-II	16.35 ± 4.12	15.60 ± 2.20	-0.644^b^	0.523
SOFA	5.30 ± 2.62	6.00 ± 2.66	0.840^b^	0.406
Medical history, *n* (%)			2.715^d^	0.267
0	6 (30)	6 (30)		
1	12 (60)	8 (40)		
≥2	2 (10)	6 (30)		
Type of ICU ward, *n* (%)			0.693^c^	0.405
Single rooms	5 (25)	2 (90)		
Shared rooms	15 (75)	18 (90)		
Mechanical ventilation duration	11.500 ± 8.62	12.350 ± 8.39	0.316^b^	0.754
Sedative medication use, *n* (%)			0.225^a^	0.974
None	16 (28.6)	15 (28.8)		
Benzodiazepines	15 (26.8)	13 (25)		
Propofol	14 (25)	12 (23.1)		
Dexmedetomidine	11 (19.6)	12 (23.1)		
Analgesic medication use, *n* (%)			0.114^a^	0.736
Yes	14 (70)	13 (65)		
None	6 (30)	7 (35)		
Anticholinergic medication use, *n* (%)			0.278^a^	0.598
Yes	3 (15)	1 (5)		
None	17 (85)	19 (95)		
Antipsychotic medication use, *n* (%)			0.000^a^	>0.999
Yes	5 (25)	4 (20)		
None	15 (75)	16 (80)		

SD: standard deviation; IQR: interquartile range; BMI: Body Mass Index; APACHE-II: Acute Physiology and Chronic Health Evaluation II; SOFA: Sequential Organ Failure Assessment; ^a^ Chi-square test; ^b^ *T*-test; ^c^ Yates’ corrected chi-square test; ^d^Fisher’s exact test.

### 4.2 Feasibility results

The total mean score of the SUS was 83.75 ± 10.21, classified as good according to the grading criteria (> 70 points). The usability and learnability dimension scores were both at the upper-middle level. The total mean score of the IPQ was 72.70 ± 7.06, at the upper-middle level. The total mean score of the SSQ was 2.06 ± 4.45, indicating fewer discomfort symptoms among participants after wearing virtual reality devices. Among the sub-dimensions, the oculomotor disturbance score was relatively higher than the other two dimensions, at 3.79 ± 8.70. The detailed results of the feasible scales in intervention group patients are shown in Appendix B: Supplementary Table S2 (Page 68).

### 4.3 Primary outcome

The positive rate of CAM-ICU in the intervention group was 5% (1/20), while that in the control group was 35% (7/20), corresponding to an absolute risk difference of −30% (95% CI: −53 to −7%). The delirium incidence (as indicated by CAM-ICU positivity) in the intervention group was lower than in the control group (χ^2^ = 3.906, *p* = 0.048). However, this pilot study was not powered to detect differences in delirium incidence, and the total number of delirium events was only eight. Consequently, the observed difference should be interpreted solely as a hypothesis-generating preliminary signal, not as confirmatory evidence of efficacy. The detailed results of delirium incidences across two groups are shown in Appendix B: Supplementary Table S3 (Page 69).

### 4.4 Secondary outcomes

Patients in the intervention group had lower delirium severity (*Z* = −2.524, 95% CI: −2.000 to 0.000, *p* = 0.012) and shorter ICU length of stay (*Z* = −2.444, 95% CI: −1.000 to 0.000, *p* = 0.015) than the control group. There was no statistically significant difference in delirium duration (*Z* = −2.420, 95% CI: 0.000 to 0.000, *p* = 0.091), hospital length of stay (*t* = 0.099, 95% CI: −2.923 to −3.223, *p* = 0.922), and time to first delirium postoperatively (*t* = −0.393, 95% CI: −56.284 to 40.713, *p* = 0.708 between the two groups). Patients in the intervention group had higher Barthel indices than the control group at ICU discharge (*t* = 2.989, 95% CI: 2.986 to 15.514, *p* = 0.005). The detailed results of the comparison between groups are shown in Appendix B: Supplementary Table S4 (Page 71).

The comparison of hospital anxiety (HADS-A) and depression (HADS-D) scores, and sleep quality (RCSQ scores) between and within groups is shown in Table 2, and cognitive function (MMSE scores) between and within groups are shown in Appendix B: Supplementary Table S5 (Page 72). There were no statistically significant differences in these scores between groups pre-intervention (*p* = 0.425, *p* = 0.353, *p* = 0.374, *p* = 0.123). The results analyzed using repeated-measures ANOVA, in conjunction with Mauchly’s test of sphericity (*p* < 0.05), showed that for HADS-A and HADS-D scores, time effects (*F* = 20.147, *p* < 0.001; *F* = 24.175, *p* < 0.001, respectively), group effects (*F* = 32.610, *p* < 0.001; *F* = 21.626, *p* < 0.001, respectively), and Time × Group interaction effects (*F* = 7.857, *p* = 0.002; *F* = 10.559, *p* < 0.001, respectively) were statistically significant. For RCSQ scores, group effects (*F* = 12.119, *p* = 0.001) and Time × Group interaction effects (*F* = 26.675, *p* < 0.001) were statistically significant.

**Table 2:** Comparison of hospital anxiety and depression (HADS-A and HADS-D) scores, sleep quality (RCSQ) scores between and within two groups (*N* = 40).

**Measures**	**T0** ^**a**^	**T1** ^**a**^	**T2** ^**a**^	**T3** ^**a**^	**Time effect**		**Group effect**		**Time × Group**		**T1**^**a**^ **vs. T0**^**a**^		**T2**^**a**^ **vs. T1**^**a**^		**T3**^**a**^ **vs. T2**^**a**^	
					***F*** *^b^*	***p***	***F*** *^b^*	***p***	***F*** *^b^*	***p***	**MD** ^c^	**p**	**MD** ^c^	**p**	**MD** ^c^	***p***
HADS-A					20.147	<0.001	32.610	<0.001	7.587	0.002						
Intervention group (*n* = 20)	9.00 ± 4.51	5.95 ± 3.28	5.60 ± 2.30	3.60 ± 1.67							−3.050	0.025	−0.350	>0.999	−2.000	<0.001
Control group (*n* = 20)	10.00 ± 3.23	11.85 ± 3.56	11.20 ± 3.55	7.10 ± 2.25							1.850	0.430	−0.650	>0.999	−4.100	<0.001
*t* ^d^	−0.807	−5.449	−5.920	−5.597												
95% CI [Lower, upper]	[−3.510, 1.518]	[−8.092, −3.708]	[−7.515, −3.685]	[−4.766, −2.234]												
*p*	0.425	<0.001	<0.001	<0.001												
HADS-D					24.175	<0.001	21.626	<0.001	10.559	<0.001						
Intervention group (*n* = 20)	7.25 ± 3.65	4.85 ± 2.46	4.80 ± 2.02	3.35 ± 1.60							−2.400	0.008	−0.050	>0.999	−1.450	0.042
Control group (*n* = 20)	8.25 ± 3.04	9.95 ± 3.49	9.55 ± 3.30	5.65 ± 1.93							1.700	0.111	−0.400	>0.999	−3.900	<0.001
*t* ^d^	−0.941	−5.349	−5.493	−4.108												
95% CI [Lower, upper]	[−3.152, 1.152]	[−7.038, 3.162]	[−6.513, −2.987]	[−3.433, −1.167]												
*p*	0.353	<0.001	<0.001	<0.001												
RCSQ					2.098	0.104	12.119	0.001	26.675	<0.001						
Intervention group (*n* = 20)	66.00 ± 12.798	75.30 ± 11.150	78.70 ± 12.127	84.30 ± 9.160							9.300	0.005	3.400	0.033	5.600	0.002
Control group (*n* = 20)	71.80 ± 10.319	68.50 ± 13.578	59.80 ± 13.057	60.80 ± 13.617							−3.300	>0.999	−8.700	0.075	1.000	>0.999
*t* ^d^	−1.578	1.731	4.743	6.404												
95% CI [Lower, upper]	[−13.242, 1.642]	[−1.153, 14.753]	[10.834, 26.966]	[16.071, 30.929]												
*p*	0.123	0.092	<0.001	<0.001												

HADS-A: Hospital Anxiety Scale; HADS-D: Hospital Depression Scale; T0: Pre-prevention; T1: Night before surgery (post-intervention); T2: ICU admission for HADS/ First night after ICU admission for RCSQ; T3: ICU discharge for HADS/ Last night before ICU discharge for RCSQ; ^a^mean ± SD; ^b^repeated-measures ANOVA; ^c^Bonferroni correction method; ^d^Independent samples *t*-test.

Regarding hospital anxiety and depression scores, following intervention pre-operatively, Patients in the intervention group had significantly lower HADS-A (*t* = −5.449, 95% CI: −8.915 to −3.485, *p* < 0.001) and HADS-D (*t* = −4.623, 95% CI: −8.915 to −3.485, *p* < 0.001) scores on the night before surgery, and ICU admission (*t* = −5.449, 95% CI: −8.092 to −3.708, *p* < 0.001; *t* = −5.349, 95% CI: −7.038 to −3.162, *p* < 0.001, respectively) than the control group. At ICU discharge, intervention group patients also had significantly lower HADS-A (*t* = −5.597, 95% CI: −4.766 to −2.234, *p* < 0.001) and HADS-D (*t* = −4.108, 95% CI: −3.433 to −1.167, *p* < 0.001) scores than the control group. Regarding MMSE scores, before the cognitive intervention, there was no statistically significant difference between groups (*t* = −0.303, 95% CI: −3.844 to 2.844, *p* = 0.764). At ICU discharge, the scores in intervention group patients were statistically significantly higher (*t* = 3.748, 95% CI: 2.074 to 7.126, *p* = 0.001). Regarding RCSQ scores, following intervention pre-operatively, there was no statistically significant difference between groups on the night before surgery (*t* = 1.731, 95% CI: −1.153 to 14.753, *p* = 0.092). After preoperative and postoperative interventions, patients in the intervention group had statistically significantly higher scores on the first night after ICU admission (*t* = 4.743, 95% CI: 10.834 to 26.966, *p* < 0.001), and on the last night before ICU discharge (*t* = 6.404, 95% CI: 16.071 to 30.929, *p* < 0.001).

The Time × Group interaction effects were statistically significant regarding hospital anxiety, depression, and sleep quality scores. For the intervention group, regarding HADS scores, the Bonferroni multiple mean comparison results showed that the HADS-A and HADS-D scores decreased after the preoperative intervention (T1-T0) (MD = −3.050, *p* = 0.025; MD = −2.400, *p* = 0.008, respectively). At ICU discharge (T3), the HADS-A and HADS-D scores were statistically significantly lower than ICU admission (T2) (MD = −2.000, *p* < 0.001; MD = −1.450, *p* = 0.042, respectively). Since neither group of patients received preoperative interventions to improve cognition, paired *t*-tests were used to analyze changes during the ICU. MMSE scores increased significantly during the ICU stay (*t* = 7.739, 95% CI: 4.122 to 7.178, *p* < 0.001), with a Cohen’s d effect size of 1.730. Regarding RCSQ scores, the Bonferroni multiple mean comparisons showed that the RCSQ scores were statistically significantly increased after preoperative intervention (T1-T0) (MD = 9.300, *p* = 0.005). After witnessing a real ICU environment nearly identical to the virtual preoperative visit, the RCSQ scores on the first night after ICU admission (T2) were statistically significantly higher than preoperative (T1) (MD = 3.400, *p* = 0.033). During the ICU stay, RCSQ scores were statistically significantly increased (T3-T2) (MD = 5.600, *p* = 0.002).

For the control group, regarding HADS scores, the Bonferroni multiple mean comparison results for HADS-A and HADS-D scores showed no statistically significant changes after preoperative intervention (T1-T0) (MD = 1.850, *p* = 0.430; MD = 1.700, *p* = 0.111, respectively). At ICU discharge (T3), the HADS-A and HADS-D scores were statistically significantly lower than ICU admission (T2) (MD = −4.100, *p* < 0.001; MD = −3.900, *p* < 0.001, respectively). MMSE scores during the ICU showed no statistically significant change (*t* = 0.735, 95% CI: −1.017 to 2.117, *p* = 0.471). Regarding RCSQ scores, the Bonferroni multiple mean comparisons showed that there were no statistically significant changes in scores after the preoperative intervention (T1-T0) (MD = −3.300, *p* > 0.999) and on the first night after ICU admission (T2-T1) (MD = 3.400, *p* = 0.033). During ICU, RCSQ scores had no statistically significant increase (T3-T2) (MD = 1.00, *p* > 0.999). The trend figures in scores for the three scales of HADS-A, HADS-D, and RCSQ across four time points of the pilot RCT are shown in Appendix B: Supplementary Figures S1, S2.

## 5 Discussion

### 5.1 Effect of the intervention on feasibility outcomes

This study demonstrated the feasibility of implementing the virtual reality-based multicomponent intervention program for cardiac surgery patients. Consistent with previous studies in cardiac surgery patients (52, 66, 91, 92), intervention group patients in this study reported higher acceptance, reflected in higher usability and learnability scores on the SUS scale. They also reported a relatively strong sense of immersion, with high IPQ scores indicating that they may have forgotten their surroundings during the VR intervention (93). This suggests that immersive VR may attenuate sensory overload and deprivation brought about by stress and noise in the ICU environment (17, 93). Additionally, we noted that almost none of the patients in this intervention group had any virtual reality use experience or had hardly even heard of virtual reality before participating in this study. That is to say, most of the intervention group patients expressed more positive evaluations of virtual reality the first time they used it without any psychological expectations. This may be explained by the fact that the immersive virtual reality system used in this study provided patients with a rich sensory stimulation and interactive experience (55, 91), which allowed patients to quickly and flexibly integrate into the immersive virtual reality environment, which was beneficial in increasing patient satisfaction, perceived benefits, and understanding of the treatment (66, 94). This finding may improve the reliability of the feasibility outcomes feedback for this virtual reality-based multicomponent intervention program; however, as suggested by Kim et al. (57), further discussion on the relationship between familiarity with virtual reality technology and intervention effects is still needed in larger studies (95).

Furthermore, we found that the mean age of intervention group patients was 63.55 ± 10.46 years, with a predominance of older patients, consistent with the mean age of cardiac surgery patients (1, 96, 97). The “digital divide” dilemma that older patients may face was previously mentioned, implying that older patients may be less interested in new technologies, including virtual reality (98). However, the results of the questionnaires in this study presented an optimistic evaluation of the virtual reality intervention by the intervention group patients, as well as a high sense of participation during the intervention period in the intervention group. Therefore, as mentioned in the study by Locke et al. (99), VR-delivered interventions may be feasible and acceptable as a delivery modality to be considered for research even in care settings with a high number of older adults, such as the cardiac surgery ward where the cardiac surgery patients selected for this study stayed preoperatively and the ICU where they were transferred postoperatively.

Moreover, we must recognize that although fewer adverse reactions were reported by patients who received virtual reality intervention in this study, this may be because of the limited sample size. Studies with larger samples need to demonstrate the adverse reactions further to obtain more convincing results. Also, data on adverse reactions to virtual reality interventions have been reported in similar studies (52, 70, 100). Still, for reasons such as limited sample size and population heterogeneity, there is no high-level evidence to summarize the details of adverse reactions to virtual reality interventions in the current cardiac surgery population. In this context, as the data from this study presented relatively high scores on the “oculomotor disturbance” dimension, this may inform further clinical research and interventions in the future.

### 5.2 Effect of the intervention on primary outcomes

The primary outcome showed a lower incidence of POD in the intervention group than in the control group. However, this pilot study was not powered to detect differences in delirium incidence, and the total number of delirium events was very limited (*n* = 8, 1 event in the intervention group and 7 events in the control group). Therefore, the observed between-group difference should be regarded solely as a hypothesis-generating preliminary signal, not as evidence of clinical efficacy. The instability of the estimate, given the small event count, further cautions against overinterpretation. This finding is consistent with Kim et al. (57), who suggested that VR-delivered multicomponent interventions may be associated with possible benefit and warrant further investigation. However, their study did not find a statistically significant effect on delirium incidence in cardiac surgery patients, and the discrepancy may be related to differences in intervention content. Specifically, the previous survey only conducted postoperative sleep intervention through a single virtual reality intervention content (virtual reality meditation), which did not highlight the advantages of multicomponent non-pharmacological prevention strategies (6, 7). Therefore, it may suggest a possible trend toward benefit in POD prevention. This study’s virtual reality intervention for POD prevention included preoperative education and a multicomponent intervention delivered via VR, targeting psychological, sleep, cognitive, and mobility domains, which was implemented daily postoperatively. It responded to the clinical evidence suggesting that POD prevention strategies should be considered as early as possible in the perioperative period encompassing both the preoperative and postoperative phases of cardiac surgery (9). Thus, this may provide preliminary data to support further investigation of this strategy for POD prevention. In addition, differences in program design, frequency, and duration of interventions may also contribute to differences in outcomes, which is also a matter of concern in future studies.

Furthermore, the prevalence of POD after cardiac surgery has been reported to be 23–52% (7–9). In this study, the prevalence of POD in the control and intervention groups was not high, at 35 and 5%, respectively. A possible explanation is that because of the nature of the study, only elective surgical cases were recruited for this study, and patients undergoing emergency surgery who were considered at high risk for POD were excluded (101). Meanwhile, the underlying health status characteristics of the participants, such as older age (≥60 years), which is considered a high-risk factor for POD (7, 102, 103); and different subtypes of delirium, such as the hypoactive subtype, which may be easily overlooked (104), may affect the statistics on the incidence of POD (105, 106). Therefore, future larger studies may be able to adopt a stratified random sampling approach to stratify different types of delirium or some high-risk factors, to increase the scientific validity of the study further.

Notably, the observed effects cannot be causally attributed to any specific component of the multicomponent intervention. Although the intervention package as a whole showed preliminary signals of benefit, the current study design does not allow us to determine which module contributed most, nor can effects be causally attributed to individual modules (e.g., Preoperative Education, Stress Response Mitigation, Circadian Reorientation, Cognitive Stimulation, or Early Mobility Promotion). Future research should consider factorial designs to test the main effects and interactions of different components, or stepwise implementation approaches to assess incremental contributions. Such designs would help identify the most active ingredients and allow for more efficient intervention optimization.

### 5.3 Effect of the intervention on secondary outcomes

The secondary outcomes showed statistically significant differences between the two groups regarding delirium severity, with a significant advantage for intervention group patients. As a clinical outcome closely related to delirium in the study, increased delirium severity and duration were associated with higher mortality and post-ICU cognitive impairment (107–112). However, probably because of the small sample, there was no statistically significant difference in delirium duration between the two groups in this study. Generally, these findings provide preliminary, hypothesis-generating signals that warrant further investigation of virtual reality multicomponent intervention on POD prevention, and suggest a possible trend that should be interpreted with caution given the small sample size. Postoperatively, the time to first POD was not statistically significantly different between intervention groups and control groups. As the occurrence of POD within 24 h postoperatively is associated with longer hospital stay lengths and higher mortality (113), this may suggest a possible benefit of virtual reality interventions, although this interpretation should remain cautious. POD is a significant independent predictor of longer ICU and total hospital length of stay (9, 114). The results of this study showed that the ICU length of stay of intervention group patients was statistically significantly lower than that of the control group, which is in line with this pattern. Whereas, the total hospital length of stay was not statistically significantly different between the two groups, which may be confounded by other influencing factors during hospitalization in the wards after transfer from the ICU, or needs further exploration in a larger sample size.

### 5.4 Effect of the intervention on anxiety and depression levels

It should be clarified that, to observe the immediate effects after the preoperative intervention and to correlate them with postoperative outcomes (115), in addition to these relevant outcomes in the ICU after the surgery, we also analyzed the anxiety and depression levels and sleep quality of the patients in both groups after the preoperative intervention.

The results of this study showed that, after experiencing virtual reality intervention, Patients in the intervention group had significantly lower levels of anxiety and depression preoperatively and during ICU hospitalization. Their scores were statistically significantly lower than those of the control group after the preoperative intervention, at the time of ICU admission and discharge. These results may be related to preoperative education through visualization enabled by virtual reality exposure, which may reduce patients’ anxiety and helplessness, as well as postoperative relaxation through virtual reality distraction therapy. Patients who are about to undergo cardiac surgery usually suffer from a high level of psychological stress and emotional burden. Reasons for this may include anticipatory anxiety about the unknown ICU environment to which they will be transferred postoperatively (81). In turn, negative preoperative emotions can continue to influence all phases of the perioperative period, adversely affecting postoperative outcomes, including POD (60, 116). Although preoperative information provision is recognized as necessary for postoperative outcomes in cardiac surgery patients (7, 67, 68, 117), a meta-analysis study found that usual preoperative educational interventions relying on healthcare professionals did not have a significant effect on decreasing cardiac surgery patients’ postoperative anxiety and depression (117). It seems that the evidence from this meta-analysis is corroborated by the findings of this study, in which the anxiety and depression levels of the control group patients were consistently high and did not change statistically significantly after the preoperative interventions and at the time of postoperative transfer to the ICU.

Compared with usual preoperative education, in this study, the virtual ICU visit realized by the virtual reality exposure method demonstrated advantages in terms of improvement in patients’ preoperative and postoperative levels of anxiety and depression. The virtual ICU visit exposed patients to the ICU to which they would be transferred postoperatively, and the virtual reality exposure method provided a safe and controlled way to visualize preoperative education (118). Patients were allowed to explore the computer-generated virtual ICU environment immersively, get a detailed first-person view of what the ICU looks and sounds like, and have equipment, POD, and treatment information explained. Vivid access to information may help reduce their anticipated fear and distress of the ICU environment (119), and was associated with lower preoperative anxiety and depression. According to emotional processing theory, preoperative virtual reality exposure may have helped patients modify their fear structure by confronting ICU-related fears and receiving POD-related information, thereby reducing anxiety when they encountered the real ICU environment after surgery (120). Meanwhile, when these patients had to deal with therapeutic care or self-care needs during their ICU stay, the preoperative virtual ICU visit experience and the knowledge learned from it may evoke a sense of security, control, and empowerment in their recovery process (121). These mechanisms may help explain the observed between-group differences in anxiety and depression during the ICU stay. Given the evidence underlying the effect of anxiety and depression levels on sleep quality in surgical patients (61, 122), we observed that after the preoperative intervention, Patients in the intervention group showed a statistically significant preliminary improvement in sleep quality on the night before surgery compared to the pre-intervention period. Although there was no statistically significant difference between the group and the control group, this result may still suggest a favorable trend.

In this study, the anxiety and depression levels of the intervention group patients at ICU discharge were statistically significantly lower than those at ICU admission, and statistically significantly lower than those in the control group. This may be related to the natural scene meditation realized through virtual reality distraction therapy during the ICU, which provided patients with visual and auditory multisensory stimulation to divert their attention. Several previous studies have supported the theory of using virtual reality distraction therapy, which includes natural scenery, music, meditation, and deep breathing, to improve negative emotions such as postoperative anxiety and depression in cardiac surgery patients (52, 92, 93, 123–126). However, we have also found that in several other studies, the implementation of virtual reality distraction therapy after cardiac surgery had a variable degree of impact on patients’ anxiety and depression levels. Differences in assessment methods may explain this, but the overall impact trend was more positive. Patients transferred to the ICU after surgery are often medically isolated and exposed to critical environments with multimodal stimulation. The combination of virtual reality technology and multisensory stimulation may distract patients from the potential for sensory overload or sensory deprivation within the ICU environment (17), while supporting recovery of emotion and attention (55, 93). The related results in this study were statistically significant improvements in anxiety and depression during the ICU stay postoperatively in patients who received virtual reality interventions. This may be explained by two complementary theories related to the restorative effects of natural environments, namely the stress recovery theory and the attention recovery theory (71, 72). VR distraction interventions may direct attention towards a relaxing immersive nature environment, reducing exposure to ICU stressors (93, 126, 127). Meanwhile, utilizing the immersive benefits of the dual combination of technology and content, providing patients with visual and auditory stimulation that facilitates more comprehensive sensory input (55, 128), may make it easier for patients to soothe cognitive and attentional fatigue during their interactions with natural meditative stimulation (93, 129–131).

### 5.5 Effect of the intervention on sleep quality

In this study, the sleep quality of intervention group patients on the night before ICU discharge was higher than that of the control group and higher than the intervention group’s score on the first ICU night. In contrast, the sleep quality of the control group did not change statistically significantly during the ICU. These findings were consistent with the findings of Lee et al. (132) and Kim et al. (57) who investigated the subjective sleep quality of postoperative cardiac patients during the ICU after using a virtual reality sleep intervention. However, these two previous studies were conducted using the virtual reality nature scene meditation method, which differed from this study. Sleep interventions to promote circadian rhythms in the ICU have been suggested to be associated with lower delirium risk (133). In light of this, and drawing on the theory of circadian care respecting biological rhythms proposed by Camargo et al. (122, 134), in this study, patients in the intervention group were provided with a virtual sleep scenario each night, where the environment for falling asleep was created by a virtual reality simulation of the changes in light that should be present in the night sky. Light is an essential element that the body uses to differentiate between day and night (135). Thus, in this situation, the statistically significant improvement in the sleep quality postoperatively may be related to the fact that the virtual reality sleep environment simulation may have reduced patients’ exposure to ICU light in the evening before falling asleep, which could affect their sleep. Meanwhile, patients’ circadian systems may also maintain a relatively stable state, and the synchronization of biorhythms and behaviors of postcardiac surgery patients in the ICU is conducive to positive postoperative outcomes (15, 136).

Furthermore, control group patients were only provided with usual care, such as eye masks and earplugs, to minimize light exposure at night, and the results showed that Patients in the intervention group had significantly better sleep quality during their stay in the ICU postoperatively than those in the control group. This may be further explained by the fact that the virtual reality sleep environment simulation additionally provided a hypnotic space conducive to focusing attention and reducing peripheral awareness (137), which could potentially help patients fall asleep and maintain a good sleep state. As in previous studies, there was an incomplete concordance between patients’ subjective and objective sleep quality outcomes. Only subjective sleep outcomes based on specialized questionnaires were reported in this study. Thus, future studies should add more objective sleep measures to clarify the effects of virtual reality interventions.

### 5.6 Effect of the intervention on cognitive function and mobility

In the 2025 update of the delirium prevention research consensus, experts identified eight research priorities for delirium prevention, with “cognitive training/exercise,” “early mobilization,” and “exercise interventions” being emphasized (31). In this study, the cognitive function of the intervention group patients at ICU discharge showed a significant improvement compared to ICU admission, and was statistically significantly higher than that of the control group; whereas the cognitive level of the control group did not change statistically significantly during their postoperative stay in the ICU. Additionally, the self-care abilities of intervention group patients at ICU discharge were significantly higher than those in the control group, which may be associated with the multicomponent intervention package on patient mobility. This finding is supported by a previous qualitative study in which patients reported that interactions with immersive virtual reality games were perceived to be associated with improved engagement in neurocognitive and motor activities, and that this fun interaction facilitated their active early postoperative rehabilitation (91). In this study, the varied interactive cognitive and early exercise games implemented through the virtual reality system may have collectively contributed to the observed favorable outcomes. Previous studies supported the impact of virtual reality technology on patients’ neurocognitive stimulation and mobility rehabilitation within the ICU (138–140). Although these studies did not focus on patients after cardiac surgery, their findings on the effects of interactive virtual reality games on cognitive and motor rehabilitation are informative. Although the study by Droc et al. (141) attempted to focus on patients undergoing major abdominal surgery, including cardiac surgery, possibly because non-interactive virtual reality rather than interactive virtual reality games were used, the results showed that such virtual reality interventions did not improve postoperative cognition. Therefore, this emphasizes that task training using virtual reality immersion and human-computer interaction may be more conducive to engaging patients actively in postoperative cognitive and motor rehabilitation. Meanwhile, according to the goal-oriented theory (142), the use of virtual reality technology as a vehicle for cognitive training and early exercise with low cognitive load may help to achieve task visualization and goal-orientation, assisting in improving the confidence and efficiency of patients’ postoperative rehabilitation in the ICU.

In the future, given the existing evidence, earlier cognitive training starting preoperatively may be considered for POD prevention in cardiac surgery patients (6, 143). Besides, although we have prepared strategies to cope with patient fatigue and safety issues that may occur during interaction with virtual reality, given the concerns raised by patients about these issues in existing studies (91), we believe in-depth interviews with patients are essential.

After discussing the findings of this study, it is important to interpret the clinical outcomes within the context of a pilot study. While the sample size (*n* = 40) was adequate for assessing feasibility and acceptability, the study was not powered to detect statistically significant differences in all secondary clinical endpoints. Therefore, the observed reductions in delirium incidence, anxiety, and depression scores should be considered preliminary. The effect sizes for these outcomes, particularly for delirium, are valuable and should be used to inform the sample size calculation for a future definitive, adequately powered randomized controlled trial.

### 5.7 Limitations

This study has several limitations. As a single-center pilot study with a small sample of elective cardiac surgery patients, its findings have limited generalizability and may be subject to selection bias. Blinding of participants and implementers was not feasible, which may have introduced performance and reporting bias. In addition, the intensity and dose of non-specific supportive care were not standardized or systematically recorded. Although all nurses received uniform training and followed a standardized clinical pathway, we did not formally audit intervention fidelity across nursing staff, nor did we quantify the exact dose or frequency of each component received by control participants. Consequently, the intervention group received more structured attention, staff interaction, and engagement time than the control group. Differences in outcomes may therefore be driven, at least in part, by attention, engagement, or rehabilitation intensity rather than by the VR technology itself. The exact time spent with staff and total duration of attention were not documented, precluding direct comparison. Future trials should consider an attention-matched control condition (e.g., a sham VR intervention or a comparable non-VR activity with equal staff interaction time) to better isolate the specific effects of the VR content. Although CAM-ICU assessments are performed routinely in clinical care and documented in medical records, these were not collected as a study-specific, standardized baseline assessment at a unified preoperative time point. The absence of a research-dedicated formal preoperative delirium and cognitive assessment may therefore have limited the accuracy of identifying incident delirium and acute changes from baseline. Future trials should include standardized baseline assessments (e.g., a study-specific preoperative CAM-ICU and, if feasible, a cognitive screening tool such as the MMSE) performed at a prespecified time point to improve diagnostic accuracy and comparability across studies. Furthermore, variability in intervention delivery and uncontrolled environmental and individual factors in the ICU may have affected intervention fidelity and consistency. Importantly, the observed between-group difference in the primary outcome should be interpreted as a preliminary signal only, given the pilot nature of the study, and the limited number of delirium events further restricts the certainty and precision of the estimated effect. Overall, these limitations highlight the exploratory nature of the findings. Regarding secondary results, the multiple secondary outcomes and repeated assessments substantially increase the risk of chance findings. Consequently, the secondary results should not be overinterpreted; they serve as preliminary signals to guide sample size calculation and endpoint selection in future larger trials. Future studies should include larger, multicenter samples, more standardized protocols, detailed recording of intervention dose and intensity, and strategies to minimize bias and confounding.

### 5.8 Recommendations or implications for practice and/or further research

This pilot randomized controlled trial is among the few exploring a virtual reality-based multicomponent intervention for preventing POD after cardiac surgery. Findings support its feasibility and acceptability, and suggest possible benefit in reducing delirium incidence and severity and improving perioperative psychological well-being, sleep, cognition, and mobility. These observations should be regarded as preliminary and require confirmation in larger trials.

## 6 Conclusion

Driven by digital health and patient-centered care, this study developed and evaluated a novel virtual reality-based multicomponent intervention for preventing POD in cardiac surgery patients—a high-risk population. The intervention integrated preoperative ICU orientation, natural scene meditation, sleep environment simulation, cognitive training, and virtual physiotherapy, offering structured perioperative support. Findings demonstrated good feasibility and acceptability, and may indicate potential benefit in reducing delirium incidence and severity and improving anxiety, depression, sleep, cognition, and mobility. However, because this was a single-center pilot trial with a small sample size and a limited number of delirium events, these findings should be interpreted cautiously. With regard to delirium incidence, the observed difference between groups should be interpreted only as a hypothesis-generating signal; the study was not powered to provide definitive evidence, and the limited number of events precludes firm conclusions. Similar caution applies to all secondary outcomes. Larger multicenter studies with long-term follow-up are needed to confirm these preliminary findings.

## Data Availability

The original contributions presented in the study are included in the article/Supplementary material, further inquiries can be directed to the corresponding author/s.
