# Supplementary material for: A virtual reality-based multicomponent intervention program for preventing postoperative delirium in the intensive care unit for cardiac surgery patients: from evidence-based design to a pilot trial
Source: Front Med (Lausanne). 2026 Jun 2;13:1815659. doi: 10.3389/fmed.2026.1815659 (PMC13268895; doi:10.3389/fmed.2026.1815659)
Supplement: Supplementary file 1 [file Table_1.pdf]

Appendix A

CONSORT checklist of information to include when reporting a randomised trial*

Section/Topic	Item No	Checklist item	Reported on item
**Title and abstract**			
	1a	Identification as a randomised trial in the title	Title
	1b	Structured summary of trial design, methods, results, and conclusions (for specific guidance see CONSORT for abstracts)	Abstract
**Introduction**			
Background and objectives	2a	Scientific background and explanation of rationale	Introduction
	2b	Specific objectives or hypotheses	Aims and Objectives
**Methods**			
Trial design	3a	Description of trial design (such as parallel, factorial) including allocation ratio	Design and Methods
	3b	Important changes to methods after trial commencement (such as eligibility criteria), with reasons	Design and Methods
Participants	4a	Eligibility criteria for participants	Design and Methods
	4b	Settings and locations where the data were collected	Design and Methods
Interventions	5	The interventions for each group with sufficient details to allow replication, including how and when they were actually administered	Design and Methods
Outcomes	6a	Completely defined pre-specified primary and secondary outcome measures, including how and when they were assessed	Design and Methods
	6b	Any changes to trial outcomes after the trial commenced, with reasons	Design and Methods
Sample size	7a	How sample size was determined	Design and

			Methods
	7b	When applicable, explanation of any interim analyses and stopping guidelines	Design and Methods
Randomisation:			Design and Methods
Sequence generation	8a	Method used to generate the random allocation sequence	Design and Methods
	8b	Type of randomisation; details of any restriction (such as blocking and block size)	Design and Methods
Allocation concealment mechanism	9	Mechanism used to implement the random allocation sequence (such as sequentially numbered containers), describing any steps taken to conceal the sequence until interventions were assigned	Design and Methods
Implementation	10	Who generated the random allocation sequence, who enrolled participants, and who assigned participants to interventions	Design and Methods
Blinding	11a	If done, who was blinded after assignment to interventions (for example, participants, care providers, those assessing outcomes) and how	Design and Methods
	11b	If relevant, description of the similarity of interventions	Design and Methods
Statistical methods	12a	Statistical methods used to compare groups for primary and secondary outcomes	Design and Methods
	12b	Methods for additional analyses, such as subgroup analyses and adjusted analyses	Design and Methods
**Results**			
Participant flow (a diagram is strongly recommended)	13a	For each group, the numbers of participants who were randomly assigned, received intended treatment, and were analysed for the primary outcome	Results
	13b	For each group, losses and exclusions after randomisation, together with reasons	Results
Recruitment	14a	Dates defining the periods of recruitment and follow-up	Results
	14b	Why the trial ended or was stopped	Results
Baseline data	15	A table showing baseline demographic and clinical characteristics for each group	Results
Numbers analysed	16	For each group, number of participants (denominator) included in each analysis and whether the analysis was by original assigned groups	Results
Outcomes and	17a	For each primary and secondary outcome, results for each group, and the estimated effect size and its precision (such as 95% confidence	Results

estimation		interval)	
	17b	For binary outcomes, presentation of both absolute and relative effect sizes is recommended	Results
Ancillary analyses	18	Results of any other analyses performed, including subgroup analyses and adjusted analyses, distinguishing pre-specified from exploratory	Results
Harms	19	All important harms or unintended effects in each group (for specific guidance see CONSORT for harms)	Results
**Discussion**			
Limitations	20	Trial limitations, addressing sources of potential bias, imprecision, and, if relevant, multiplicity of analyses	Discussion
Generalisability	21	Generalisability (external validity, applicability) of the trial findings	Discussion
Interpretation	22	Interpretation consistent with results, balancing benefits and harms, and considering other relevant evidence	Discussion
**Other information**			
Registration	23	Registration number and name of trial registry	Design and Methods
Protocol	24	Where the full trial protocol can be accessed, if available	Design and Methods
Funding	25	Sources of funding and other support (such as supply of drugs), role of funders	Title page

*We strongly recommend reading this statement in conjunction with the CONSORT Explanation and Elaboration for important clarifications on all the items. If relevant, we also recommend reading CONSORT extensions for cluster randomised trials, non-inferiority and equivalence trials, non-pharmacological treatments, herbal interventions, and pragmatic trials. Additional extensions are forthcoming: for those and for up to date references relevant to this checklist, see www.consort-statement.org.
