# Supplementary material for: A virtual reality-based multicomponent intervention program for preventing postoperative delirium in the intensive care unit for cardiac surgery patients: from evidence-based design to a pilot trial
Source: Front Med (Lausanne). 2026 Jun 2;13:1815659. doi: 10.3389/fmed.2026.1815659 (PMC13268895; doi:10.3389/fmed.2026.1815659)
Supplement: Supplementary file 2 [file Data_Sheet_1.docx]

**Appendix B**

**LEGENDS**

**Tables**

**Table S1.** Detailed description of the five-component virtual reality intervention.

**Table S2.**Scores of System Usability Scale, Technology Acceptance Scale, Igroup Presence Questionnaire, Information Availability Satisfaction Scale, Simulator Sickness Questionnaire (N = 20).

**Table S3.** Comparison of delirium incidences across two groups (N = 40).

**Table S4.** Between-group differences in delirium-related and clinical outcomes (N = 40).

Note: ^a^ mean ± SD; ^b^ median (IQR).

**Table S5.** Comparison of cognitive level (MMSE) scores between and within two groups (N = 40).

Note: MMSE: Mini-mental State Examination; T0: Pre-prevention; T1: ICU admission; T2: ICU discharge; ^a^ mean ± SD; ^b^ Independent samples t-test；^c^ Paired samples t-test to compare the cognitive function scores between T1 and T2.

**Figures**

**Figure S1 (a).** Hospital anxiety scores (HADS-A) for two groups at four time points.

Note: HADS-A: Hospital anxiety scores; T0: Pre-prevention; T1: Night before surgery (post-intervention); T2: ICU admission; T3: ICU discharge.

**Figure S1 (b).** Hospital depression scores (HADS-D) for two groups at four time points.

Note: HADS-D: Hospital depression scores; T0: Pre-prevention; T1: Night before surgery (post-intervention); T2: ICU admission; T3: ICU discharge.

**Figure S2.** Sleep quality (RCSQ) scores for two groups at four time points.

Note: RCSQ: Richards-Campbell Sleep Questionnaire; T0: Pre-prevention; T1: Night before surgery (post-intervention); T2: First night after ICU admission; T3: Last night before ICU discharge.

**Table S1.** Detailed description of the five-component virtual reality intervention.

| Module Name & Primary Aim | Core Content & Key Activities | Technical Specifications & Interactive Logic | Dosage & Dynamic Adjustment Rules |
| --- | --- | --- | --- |
| 1. Preoperative Education Aim: To reduce preoperative anxiety and enhance preparedness by familiarizing patients with the postoperative ICU environment and care processes. | Content: A simulated virtual ICU tour. Patients can visually explore a typical ICU bed space and encounter virtual representations of common equipment (ventilator, monitor, drains) and staff. Key Activities: Listening to a narrated introduction about the purpose of equipment, routine nursing procedures (e.g., suctioning, turning), and an explanation of postoperative delirium (POD) as a potential complication. | Media Type: Real-time rendered 3D environment. Interactivity: Limited. Navigation is via head-gaze to look around the static scene. Informational hotspots are activated by gazing, triggering audio explanations. Progression: Linear, fixed sequence of explanations. | Duration: 5-6 minutes, administered once preoperatively. Adjustment: None. Content is standardized for all patients. |
| 2. Stress Response Mitigation Aim: To induce psychophysiological relaxation and mitigate stress/anxiety through immersive nature exposure and guided mindfulness. | Content: Serene, dynamic natural environments (e.g., forest, beach). Key Activities: Passive immersion combined with audio-delivered, gentle female-voiced mindfulness meditation scripts focusing on breath awareness and body scan. | Media Type: 360° video or rendered environment with dynamic elements (e.g., changing light, moving leaves). Interactivity: Non-interactive. Patients observe and listen. Progression Logic: Scene parameters (e.g., light intensity, virtual "wind" speed/sound) can automatically soften as the session progresses to deepen relaxation. | Duration: 20-25 minutes per session. Adjustment: The system can subtly reduce environmental stimulation over time based on a preset timeline. Patient can pause or stop at any time. |
| 3. Circadian Reorientation Aim: To promote sleep-wake cycle regulation and provide a calming nocturnal stimulus using immersive sleep-themed environments. | Content: Two themes: "Stargazing" (static starry sky) and "Interstellar Travel" (slow motion through a starfield). Key Activities: Passive viewing accompanied by specially composed, hypnotic, and rhythmic ambient music designed to facilitate relaxation and sleepiness. | Media Type: High-fidelity 720° panoramic images or very slow-motion 360° video. Interactivity: Non-interactive. Progression Logic: "Interstellar Travel" provides a gentle, continuous visual flow to guide attention. | Duration: 10-15 minutes, typically offered in the evening. Adjustment: None. The patient or researcher selects the theme prior to the session. |
| 4. Cognitive Stimulation Aim: To provide targeted, interactive cognitive training to engage attention, memory, and executive functions, potentially enhancing cognitive reserve. | Content: A suite of gamified cognitive tasks adapted for VR (e.g., Stroop task, virtual object matching, spatial memory games, simple arithmetic tasks). Key Activities: Active participation in tasks requiring selection, matching, recall, or calculation via controller interaction. | Media Type: Real-time rendered 3D game environments. Interactivity: High. Direct interaction via handheld controllers for selection and manipulation. Progression Logic: Difficulty adjusts automatically based on performance (e.g., faster stimulus presentation, more complex tasks after consecutive correct answers). | Duration:15 minutes per session. Adjustment: Built-in adaptive algorithm modifies task difficulty in real-time. System provides positive audio feedback for correct responses. |
| 5. Early Mobility Promotion Aim: To encourage safe, early active limb movement through engaging virtual physiotherapy, promoting functional recovery. | Content: Virtual environments that guide functional upper limb exercises (e.g., reaching for virtual objects on a shelf, stirring a virtual pot). Key Activities: Patients mimic the movements of a virtual coach avatar. Inertial sensors in the VR controllers provide real-time motion capture. | Media Type: Real-time rendered 3D environment. Interactivity: High. Movement-based interaction with precise tracking. Progression Logic: Exercise sequence is fixed per session but progresses in range of motion or complexity across sessions as tolerated. | Duration: 10-15 minutes per session. Adjustment: Number of repetitions can be suggested potential reduction by the supervising nurse based on patient fatigue. The system provides visual and verbal form correction prompts. |
| General Platform Specifications | Headset: Pico Neo 3 (Standalone). Development Platform: Unity3D (v2021.3 LTS). Safety Feature: A prominent "Exit" button is always accessible on the virtual wrist menu and the physical controller. |  |  |

**Table S2.** Scores of System Usability Scale, Technology Acceptance Scale, Igroup Presence Questionnaire, Information Availability Satisfaction Scale, Simulator Sickness Questionnaire (N = 20).

| **Scales** | **Range** | **Total score (Mean ± SD)** |
| --- | --- | --- |
| **System Usability Scale (SUS)** | 0-100 | 83.750±10.212 |
| **Usablility** | 0-100 | 86.25±10.797 |
| **Learnability** | 0-100 | 73.75±18.541 |
| I think that I would like to use this system frequently | 0-4 | 3.70±0.571 |
| I found the interface unnecessarily complex | 0-4 | 3.20±0.696 |
| I thought the system was easy to use | 0-4 | 3.65±0.489 |
| I think that I would need the support of a technical person to be able to use this system | 0-4 | 2.75±0.851 |
| I found the various functions in this system were well integrated | 0-4 | 3.35±0.671 |
| I thought there was too much inconsistency in this system | 0-4 | 3.15±0.745 |
| I would imagine that most people would learn to use this system very quickly | 0-4 | 3.45±0.759 |
| I found the system very cumbersome to use | 0-4 | 3.50±0.607 |
| I felt very confident using the system | 0-4 | 3.60±0.598 |
| I needed to learn a lot of things before I could get going with this system. | 0-4 | 3.15±0.813 |
| **Igroup Presence Questionnaire (IPQ)** | 13-91 | 72.70±7.064 |
| Spatial presence (SP) | 5-35 | 27.75±3.754 |
| Involvement (INV) | 4-28 | 22.25±2.613 |
| Reality (REAL) | 3-21 | 16.65±3.133 |
| **Simulator Sickness Questionnaire (SSQ)** | 0-179.52 | 2.057±4.454 |
| Nausea | 0-200.34 | 1.4310±4.669 |
| Oculomotor disturbance | 0-159.18 | 3.7900±8.695 |
| Disorientation | 0-292.32 | 2.7840±7.282 |

**Table S3.** Comparison of delirium incidences across two groups (N = 40).

| **Groups** | N | **Positive n (%)** | **Negative n (%)** | **χ^2^** | **p** |
| --- | --- | --- | --- | --- | --- |
| **Intervention group** | 20 | 19 (95.0) | 1 (5.0) | 3.906 | 0.048 |
| **Control group** | 20 | 13 (65.0) | 7 (35.0) |  |  |

**Table S4.** Between-group differences in delirium-related and clinical outcomes (N = 40).

| **Outcomes** | **Intervention group (n=20)** | **Control group (n=20)** | **t/Z value** | **95 % CI [Lower, upper]** | **p** |
| --- | --- | --- | --- | --- | --- |
| **Delirium severity**^b^ | 0.00 (0.00, 1.00) | 1.50 (0.00, 5.00） | -2.524 | [−2.000, 0.000] | 0.012 |
| **Delirium duration (h) ^b^** | 0.000 (0.000, 0.000) | 0.000 (0.000, 15.125) | -2.420 | [0.000, 0.000] | 0.091 |
| **Time to first delirium postoperatively (h) ^a^** | 28.000±0.000 | 20.214±7.008 | -0.393 | [−56.284, 40.713] | 0.708 |
| **ICU length of stay (day) ^b^** | 2.00 (2.00, 2.75) | 3.00 (2.00, 4.75) | -2.444 | [−1.000, 0.000] | 0.015 |
| **Hospital length of stay (day) ^a^** | 16.60±4.762 | 16.45±4.839 | 0.099 | [−2.923, −3.223] | 0.922 |
| **Barthel (ICU transfer) ^a^** | 51.75±8.472 | 42.50±10.942 | 2.989 | [2.986, 15.514] | 0.005 |

Note: ^a^ mean ± SD; ^b^ median (IQR).

**Table S5.** Comparison of cognitive level (MMSE) scores between and within two groups (N = 40).

| **Group** | **T0^a^** | **T1^a^** | **T2^a^** | **t^b^** | **95 % CI [Lower, upper]** | **p** | **Effect size^b^** |
| --- | --- | --- | --- | --- | --- | --- | --- |
| **Intervention group (n=20)** | 27.20±2.24 | 21.65±3.84 | 27.30±2.11 | 7.739 | [4.122, 7.178] | <0.001 | 1.730 |
| **Control group (n=20)** | 26.40±3.28 | 22.15±6.31 | 22.70±5.07 | 0.735 | [-1.017, 2.117] | 0.471 | 0.164 |
| **t^c^** | 0.900 | -0.303 | 3.748 |  |  |  |  |
| **p** | 0.374 | 0.764 | 0.001 |  |  |  |  |

Note: MMSE: Mini-mental State Examination; T0: Pre-prevention; T1: ICU admission; T2: ICU discharge; ^a^ mean ± SD; ^b^ Independent samples t-test；^c^ Paired samples t-test to compare the cognitive function scores between T1 and T2.

**
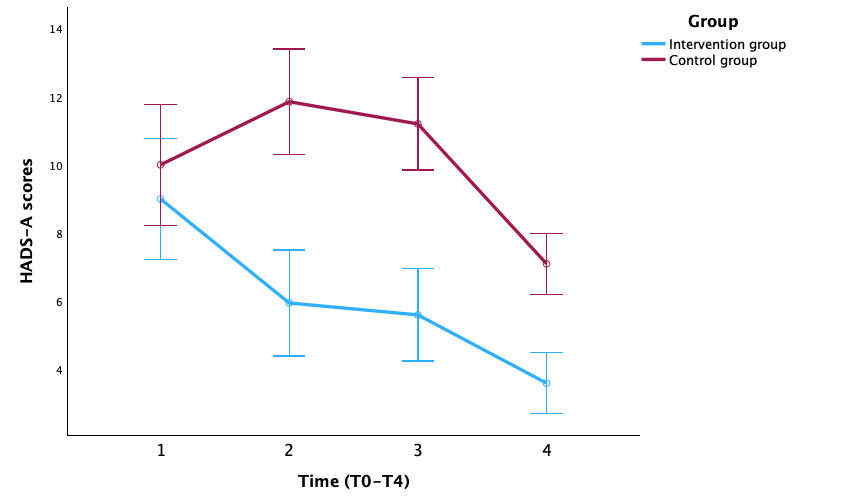
**

**Figure S1 (a).** Hospital anxiety scores (HADS-A) for two groups at four time points.

Note: HADS-A: Hospital anxiety scores; T0: Pre-prevention; T1: Night before surgery (post-intervention); T2: ICU admission; T3: ICU discharge.


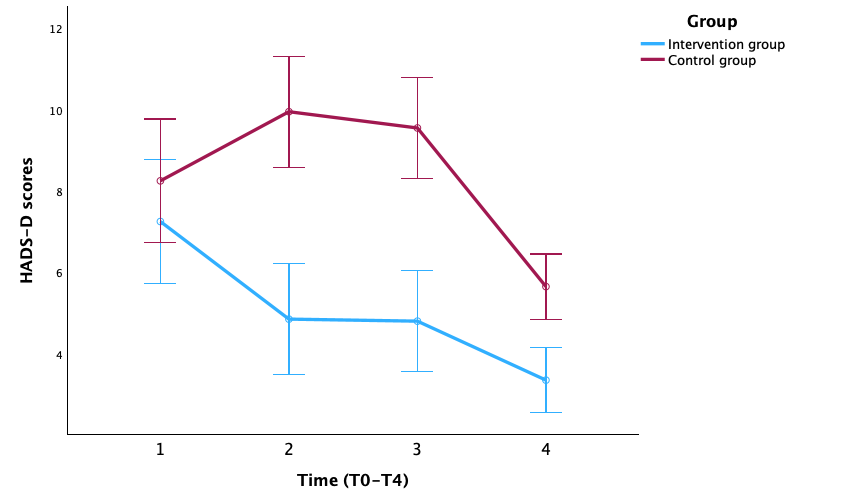


**Figure S1 (b).** Hospital depression scores (HADS-D) for two groups at four time points.

Note: HADS-D: Hospital depression scores; T0: Pre-prevention; T1: Night before surgery (post-intervention); T2: ICU admission; T3: ICU discharge.

**
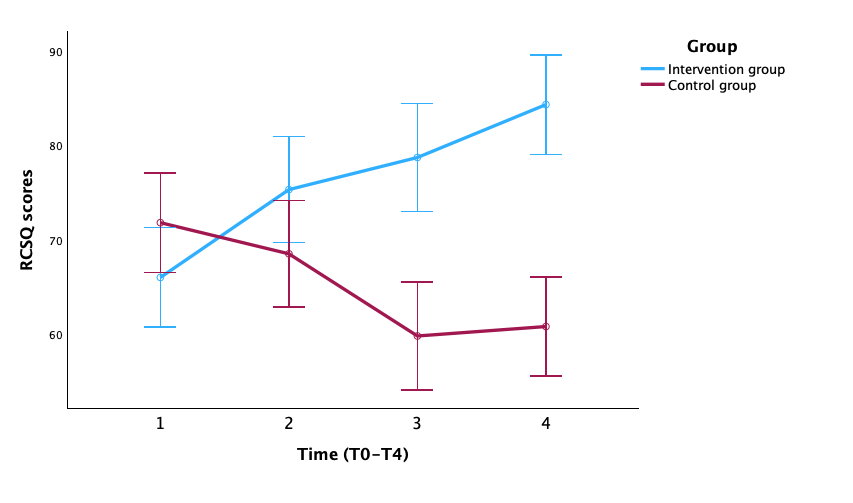
Figure S2.** Sleep quality (RCSQ) scores for two groups at four time points.

Note: RCSQ: Richards-Campbell Sleep Questionnaire; T0: Pre-prevention; T1: Night before surgery (post-intervention); T2: First night after ICU admission; T3: Last night before ICU discharge.
